# Prevalence, sex differences, and implications of pulmonary hypertension in patients with apical hypertrophic cardiomyopathy

**DOI:** 10.3389/fcvm.2023.1288747

**Published:** 2024-01-11

**Authors:** Vidhu Anand, Megan K. Covington, Ushasi Saraswati, Christopher G. Scott, Alexander T. Lee, Robert P. Frantz, Nandan S. Anavekar, Jeffrey B. Geske, Adelaide M. Arruda-Olson, Kyle W. Klarich

**Affiliations:** ^1^ Department of Cardiovascular Medicine, Mayo Clinic, Rochester, MN, United States; ^2^ Department of Quantitative Health Sciences, Mayo Clinic, Rochester, MN, United States

**Keywords:** apical hypertrophic cardiomyopathy, pulmonary hypertension, pulmonary artery systolic pressure, sex differences, all-cause mortality

## Abstract

**Introduction:**

Apical hypertrophic cardiomyopathy (ApHCM) is a subtype of hypertrophic cardiomyopathy (HCM) that affects up to 25% of Asian patients and is not as well understood in non-Asian patients. Although ApHCM has been considered a more “benign” variant, it is associated with increased risk of atrial and ventricular arrhythmias, apical thrombi, stroke, and progressive heart failure. The occurrence of pulmonary hypertension (PH) in ApHCM, due to elevated pressures on the left side of the heart, has been documented. However, the exact prevalence of PH in ApHCM and sex differences remain uncertain.

**Methods:**

We sought to evaluate the prevalence, risk associations, and sex differences in elevated pulmonary pressures in the largest cohort of patients with ApHCM at a single tertiary center. A total of 542 patients diagnosed with ApHCM were identified using ICD codes and clinical notes searches, confirmed by cross-referencing with cardiac MRI reports extracted through Natural Language Processing and through manual evaluation of patient charts and imaging records.

**Results:**

In 414 patients, echocardiogram measurements of pulmonary artery systolic pressure (PASP) were obtained at the time of diagnosis. The mean age was 59.4 ± 16.6 years, with 181 (44%) being females. The mean PASP was 38 ± 12 mmHg in females vs. 33 ± 9 mmHg in males (*p* < 0.0001). PH as defined by a PASP value of > 36 mmHg was present in 140/414 (34%) patients, with a predominance in females [79/181 (44%)] vs. males [61/233 (26%), *p* < 0.0001]. Female sex, atrial fibrillation, diagnosis of congestive heart failure, and elevated filling pressures on echocardiogram remained significantly associated with PH (PASP > 36 mmHg) in multivariable modeling. PH, when present, was independently associated with mortality [hazard ratio 1.63, 95% CI (1.05–2.53), *p* = 0.028] and symptoms [odds ratio 2.28 (1.40, 3.71), *p* < 0.001].

**Conclusion:**

PH was present in 34% of patients with ApHCM at diagnosis, with female sex predominance. PH in ApHCM was associated with symptoms and increased mortality.

## Introduction

Apical hypertrophic cardiomyopathy (ApHCM) is a subtype of hypertrophic cardiomyopathy (HCM) with hypertrophy localized to the left ventricular (LV) apex. The estimated prevalence rate among patients with HCM varies from 25% in the Asian population to 1%–10% in the non-Asian population (1, 2). Patients with ApHCM have a widely variable clinical presentation, ranging from being asymptomatic with a normal lifespan to being symptomatic with dyspnea, reduced exercise capacity, chest pain, atrial fibrillation, heart failure, thromboembolic events, and ventricular arrhythmias, or experiencing sudden cardiac death (2–5). The development of an apical pouch and aneurysm is known to have negative prognostic effect, predisposing to both ventricular arrhythmias and intracardiac thrombus formation (4, 6, 7).

Pulmonary hypertension (PH) is prevalent in HCM, with an estimated prevalence rate of 38% by echocardiography (8). It has been shown that older age and systolic dysfunction are independent risk factors for developing PH in HCM (9). Another case–control study found female sex, moderate or greater mitral regurgitation, and atrial fibrillation as independent risk factors for PH beyond the effect of age (10). The prevalence of PH was similar in obstructive and non-obstructive HCM in one study (8), and it was higher in obstructive and end-stage HCM in another (9). Despite the slight differences in prevalence, PH was an independent predictor of mortality in patients with both obstructive and non-obstructive HCM (8, 9, 11). A high prevalence of PH by echocardiography in ApHCM with a female predominance has been described, but the study was not designed to evaluate the risk associations and implications of PH (7). Therefore, in this study, we sought to evaluate the prevalence, sex differences, and risk associations of PH in ApHCM and to assess the prognostic implications of PH in a large cohort of patients with isolated ApHCM at a single referral center in the United States.

## Methods

### Patient population

This study was approved by the institutional review board and deemed exempt. All patients provided consent to the use of their data for research. A total of 542 patients diagnosed with ApHCM were identified by using ICD-9/10 codes and a search of text contained within clinical notes. The diagnosis was further confirmed by cross-referencing with cardiac MRI reports extracted through Natural Language Processing, as well as through manual review of patient charts, echocardiograms, and cardiac MRIs from January 1999 to May 2018. ApHCM was defined on imaging as the presence of apical wall thickness measuring ≥15 mm or ≥13 mm in individuals with a positive family history or positive genetic mutation. Patients with other patterns of LV hypertrophy without clear apical predominance (reverse curve, sigmoid, neutral septum), infiltrative cardiomyopathies including amyloidosis, Fabry disease, or secondary causes of LV hypertrophy including hypertensive heart disease were excluded. Patients with eosinophilic heart disease/eosinophilic myocarditis were also excluded after conducting a careful review of their cardiac MRI and pathology results when available. While patients with hypertensive heart disease were excluded, those with ApHCM and a concomitant diagnosis of hypertension were not excluded (12). All included patients underwent a comprehensive transthoracic echocardiography (TTE), and the initial study that diagnosed ApHCM was used for analysis. Baseline characteristics recorded at the time of index TTE, including demographic data, diagnosis of congestive heart failure and comorbidities determined from ICD codes, and Charlson comorbidity index, were extracted from the electronic medical records. The New York Heart Association (NYHA) functional class and the presence of symptoms (NYHA functional classes II–IV) were manually abstracted by review of charts. The vital status was retrieved using the Mayo Clinic records. Patients not known to be deceased were censored at the date of the last follow-up.

### Echocardiography

Echocardiographic assessment was reported by Level 3 trained echocardiographer, and data included LV linear dimensions and ejection fraction, mitral inflow early/ late diastolic velocity (E/A), mitral annular early tissue Doppler velocity (e'), medial E/e' (surrogate of elevated filling pressures), left atrial volume index, pulmonary artery systolic pressure (PASP), estimated right atrial pressure, RV size, and RV systolic function. Pulmonary hypertension was defined as none (PASP < 36 mmHg), mild-to-moderate (PASP 36–59 mmHg), and severe (PASP ≥ 60 mmHg). The PASP was evaluated from the highest (or average of five cardiac cycles in patients with atrial fibrillation or significant respiratory variation) and most complete signal of tricuspid regurgitation from the RV inflow, parasternal short axis, and apical views using modified Bernoulli equation as 4*v*^2 ^+ estimated right atrial pressure, where *v* is the velocity of the tricuspid regurgitation jet in m/s. The right atrial pressure was estimated based on the size and collapse of the inferior vena cava as follows: 5 mmHg when it displayed both normal size and collapse, 10 mmHg when it was either enlarged or had reduced collapse, 15 mmHg when it was both enlarged and had reduced collapse, and 20 mmHg when it was enlarged with no collapse. The RV was considered enlarged if its size exceeded mild enlargement, and its function was categorized as reduced if it was more than mildly reduced. RV size and function assessments, as specified in the echocardiographic reports, primarily included qualitative evaluations and, when available, quantitative measures (available in less than 50% of the patients).

### Outcomes

The primary outcome was the prevalence of PH in patients with ApHCM, sex differences in the same, and impact of PH on all-cause mortality. The secondary outcomes were the risk associations of PH and the impact of PH on symptoms.

### Statistical analysis

Data are presented as frequencies and percentages for categorical variables, and as mean with standard deviation (SD) for approximately normally distributed continuous variables or median and quartiles for those that were not. Chi-square test was used to compare the categorical variables and *t*-test or Kruskal–Wallis for continuous variables, as appropriate. The survival curves were constructed using the Kaplan–Meier method, and the groups of PH patients were compared using the log-rank test. Cox proportional hazards regression was used to examine the association between PH and all-cause mortality after adjusting for other factors noted to be significant in the univariate analysis. These results are presented as hazard ratios (HR) with 95% confidence intervals (CI). Multivariable models were created using backward selection starting from those variables that were significant in the univariate analyses. Logistic regression was used to examine the factors associated with PH and to assess the association between PH and symptoms, and these results are presented in terms of odds ratios (OR) and 95% CI. The model assumptions were checked graphically to ensure that no violations were noted. The risk for mortality by PASP was illustrated graphically after fitting the PASP using penalized smoothing splines, and Youden's *J* index was used to identify the optimal cut point for this association. All analyses were performed using SAS version 9.4 (SAS Institute, Inc. Cary, NC, USA), and a *p*-value of < 0.05 was considered statistically significant.

## Results

**Figure 1:**
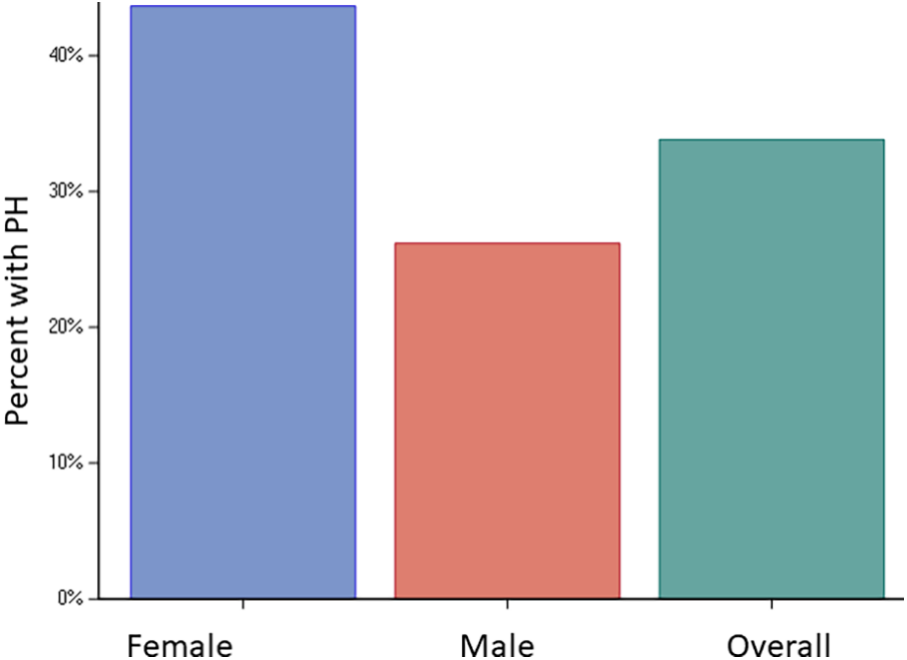
Prevalence of PH. Bar graphs showing prevalence of PH in the entire cohort (34%) and in females (44%) vs. males (26%), *p* < 0.001.

The study cohort consisted of 414/542 (76%) patients who had a sufficient tricuspid regurgitation Doppler signal for estimating PASP. The mean age was 59.4 ± 16.6 years, with 181 (44%) being females. Among the patients, 184 (47%) had hypertension, 41 (10%) had diabetes, 39 (10%) had a diagnosis of coronary artery disease, and 21 (5%) had prior myocardial infarction (Table 1). In terms of history of ventricular arrhythmias, 34 (9%) patients had ventricular tachycardia, seven (2%) had ventricular fibrillation, and three (1%) had prior cardiac arrest. The baseline characteristics are presented in Table 1. Pulmonary hypertension was present in 140 (34%) patients, with a higher prevalence in females (*n* = 79/181, 44%) than males (*n* = 61/233, 26%), *p* < 0.001 (Figure 1). Severe PH was present in 10 (2%) patients with a higher prevalence in females than males [seven (4%) vs. three (1%), *p* = 0.001]. The baseline characteristics of males and females are presented separately in Supplementary Table S1. The univariate factors associated with PH were found to be age, female sex, moderate or greater MR, larger left atrial volume index, higher medial E/e', atrial fibrillation, diagnosis of congestive heart failure, and Charlson comorbidity index. MR etiology included annular dilatation (atrial functional MR) in 11 patients, mitral valve prolapse in one patient, and annular dilatation along with significant mitral annulus calcification in one patient. In the multivariable model, female sex [OR 1.88 (1.17, 3.04), *p* = 0.009], medial E/e' > 15 [OR 2.06 (1.22, 3.49), *p* = 0.007], atrial fibrillation [OR 2.09 (1.09, 4.01), *p* = 0.026], and a diagnosis of congestive heart failure [OR 2.48 (1.28, 4.81), *p* = 0.007] remained independently associated with PH (Table 2). In another model, when left atrial volume index was substituted for medial E/e’, it was also observed to have a statistically significant correlation (Supplementary Table S2).

**Table 1:** Baseline characteristics.

Variables	Total (*N* = 414)	No PH (*N* = 274)	PH (*N* = 140)	*p*-value
Age	59.4 (16.63)	56.7 (16.62)	64.7 (15.39)	<0.001
Female sex, *n* (%)	181 (43.7%)	102 (37.2%)	79 (56.4%)	<0.001
Race, *n* (%)				0.708
White	341 (82.4%)	226 (82.5%)	115 (82.1%)	
Black	20 (4.8%)	13 (4.7%)	7 (5.0%)	
Asian	17 (4.1%)	12 (4.4%)	5 (3.6%)	
Choose not to disclose	4 (1.0%)	4 (1.5%)	0 (0.0%)	
Other	9 (2.2%)	5 (1.8%)	4 (2.9%)	
Unknown	23 (5.6%)	14 (5.1%)	9 (6.4%)	
Body mass index	28.3 (5.20)	28.4 (5.25)	28.1 (5.11)	0.655
Heart rate (*n* = 408)	63.9 (11.97)	63.5 (11.49)	64.8 (12.85)	0.299
Systolic blood pressure (*n* = 410)	124.2 (19.41)	122.6 (18.96)	127.5 (19.97)	0.017
Atrial fibrillation, *n* (%) (*n* = 396)	58 (14.6%)	25 (9.5%)	33 (25.0%)	<0.001
Prior myocardial infarction, *n* (%) (*n* = 395)	21 (5.3%)	9 (3.5%)	12 (8.9%)	0.023
Congestive heart failure, *n* (%) (*n* = 395)	57 (14.4%)	21 (8.1%)	36 (26.7%)	<0.001
Ventricular tachycardia, *n* (%) (*n* = 395)	34 (8.6%)	25 (9.6%)	9 (6.7%)	0.322
Ventricular fibrillation, *n* (%) (*n* = 395)	7 (1.8%)	5 (1.9%)	2 (1.5%)	0.752
Cardiac arrest, *n* (%) (*n* = 395)	3 (0.8%)	1 (0.4%)	2 (1.5%)	0.234
PVC, *n* (%) (*n* = 395)	29 (7.3%)	23 (8.8%)	6 (4.4%)	0.112
Transient ischemic attack, *n* (%) (*n* = 395)	22 (5.6%)	12 (4.6%)	10 (7.4%)	0.251
Ischemic stroke, *n* (%) (*n* = 395)	18 (4.6%)	14 (5.4%)	4 (3.0%)	0.274
Hypertension, *n* (%) (*n* = 395)	184 (46.6%)	113 (43.5%)	71 (52.6%)	0.084
Diabetes, *n* (%) (*n* = 395)	41 (10.4%)	25 (9.6%)	16 (11.9%)	0.489
Chronic kidney disease, *n* (%) (*n* = 395)	10 (2.5%)	7 (2.7%)	3 (2.2%)	0.778
Lung disease, *n* (%) (*n* = 395)	50 (12.7%)	27 (10.4%)	23 (17.0%)	0.059
Coronary artery disease, *n* (%) (*n* = 395)	39 (9.9%)	24 (9.2%)	15 (11.1%)	0.552
Charlson Index, median (Q1, Q3) (*n* = 395)	2 (0, 6)	1 (0, 4.5)	2 (1, 7)	0.002
LV ejection fraction	66.8 (6.40)	67.0 (5.51)	66.5 (7.86)	0.450
Diastolic dysfunction grade, *n* (%) (*n* = 126)				0.006
Normal	14 (11.1%)	12 (15.2%)	2 (4.3%)	
1 (normal filling pressures)	21 (16.7%)	17 (21.5%)	4 (8.5%)	
1a (mildly elevated filling pressures with A-predominant mitral inflow)	13 (10.3%)	11 (13.9%)	2 (4.3%)	
2 (mild–moderately elevated filling pressures)	32 (25.4%)	18 (22.8%)	14 (29.8%)	
3+ (severely elevated filling pressures)	7 (5.6%)	2 (2.5%)	5 (10.6%)	
Indeterminate	39 (31.0%)	19 (24.1%)	20 (42.6%)	
Medial e’ (*n* = 366)	0.06 (0.07)	0.07 (0.09)	0.05 (0.02)	<0.001
Medial E/e’, median (*Q*1, *Q*3)	12 (10, 16)	12 (10, 15)	14 (12, 18)	<0.001
Medial E/e’>15, *n* (%)	108 (26.1%)	55 (20.1%)	53 (37.9%)	<0.001
E/A, median (Q1, Q3) (*n* = 340)	1.3 (0.9, 1.8)	1.2 (0.9, 1.8)	1.5 (1.0, 2.0)	0.009
RV S’ (*n* = 200)	0.12 (0.03)	0.12 (0.02)	0.12 (0.04)	0.893
TAPSE (*n* = 59)	20.0 (5.38)	20.0 (3.50)	20.0 (7.17)	0.962
PASP	35.1 (10.56)	29.0 (4.24)	47.0 (8.89)	<0.001
PASP, *n* (%)				<0.001
<=36	274 (66.2%)	274 (100.0%)	0 (0.0%)	
37–60	130 (31.4%)	0 (0.0%)	130 (92.9%)	
>60	10 (2.4%)	0 (0.0%)	10 (7.1%)	
RV enlargement present, *n* (%) (*n* = 242)	14 (5.8%)	3 (2.0%)	11 (12.0%)	0.001
RV function reduced, *n* (%) (*n* = 73)	16 (21.9%)	3 (7.3%)	13 (40.6%)	<0.001
Right atrial pressure, median (Q1, Q3) (*n* = 407)	5 (5, 5)	5 (5, 5)	10 (5, 10)	<0.001
LA volume index (*n* = 349)	41.3 (16.21)	36.4 (11.07)	51.5 (20.06)	<0.001
Max. instantaneous intracavitary gradient (*n* = 102)	27.3 (16.75)	27.6 (18.24)	26.7 (13.13)	0.803
≥moderate mitral regurgitation, *n* (%) (*n* = 405)	13 (3.2%)	3 (1.1%)	10 (7.3%)	<0.001

PVC, premature ventricular contraction; LV, left ventricle; E/e’, mitral early inflow diastolic/mitral annulus early tissue Doppler velocity; E/A, mitral inflow early/late diastolic velocity; PASP, pulmonary artery systolic pressure; RV, right ventricle; LA, left atrium.

**Table 2:** Univariate and multivariable analyses of factors associated with PH.

	Univariate logistic regression		Multivariable logistic regression (*N* missing = 45)	
Variable	Odds ratio (95% CI)	*p*-value	Odds ratio (95% CI)	*p*-value
Age	1.03 (1.02, 1.05)	<0.001	1.02 (1.00, 1.03)	0.073
Female sex	2.18 (1.44, 3.30)	<0.001	1.88 (1.17, 3.04)	0.009
Coronary artery disease	1.23 (0.62, 2.43)	0.553		
Hypertension	1.44 (0.95, 2.19)	0.085		
Chronic kidney disease	0.82 (0.21, 3.23)	0.778		
Diabetes	1.26 (0.65, 2.46)	0.490		
Lung disease	1.77 (0.97, 3.23)	0.062		
≥Moderate MR	6.95 (1.88, 25.69)	0.004	3.40 (0.81, 14.23)	0.094
LV ejection fraction	0.99 (0.96, 1.02)	0.450		
Medial E/e’ > 15	2.43 (1.54, 3.81)	<0.001	2.06 (1.22, 3.49)	0.007
LA volume index	1.08 (1.06, 1.10)	<0.001		
LVOT MIG	1.02 (0.99, 1.05)	0.212		
Intracavitary MIG	0.997 (0.97, 1.02)	0.801		
Atrial fibrillation	3.19 (1.80, 5.64)	<0.001	2.09 (1.09, 4.01)	0.026
Congestive heart failure	4.14 (2.30, 7.44)	<0.001	2.48 (1.28, 4.81)	0.007
Charlson comorbidity index	1.09 (1.02, 1.16)	0.008	1.06 (0.99, 1.14)	0.103

MR, mitral regurgitation; LV, left ventricle; E/e’, mitral early inflow diastolic/mitral annulus early tissue Doppler velocity; LA, left atrial; LVOT, left ventricular outflow tract; MIG, maximal instantaneous gradient.

**Figure 2:**
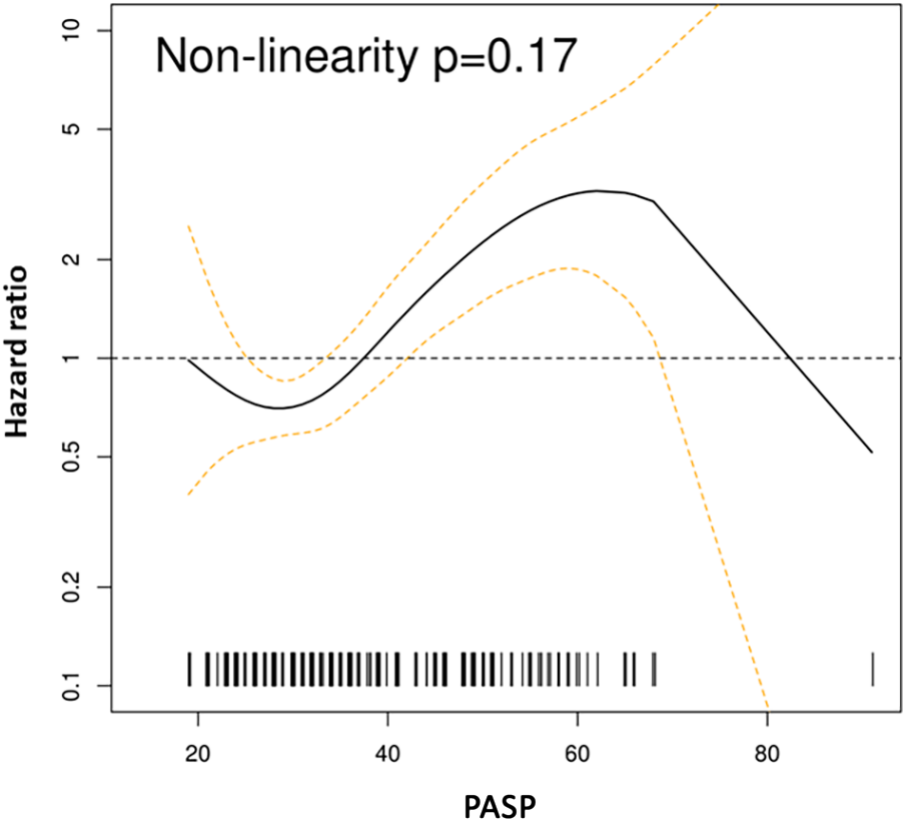
Spline curves for PASP cutoff. Spline curves demonstrate mortality risk across the range of measured PASP in the whole cohort. The risk increased for PASP > 35–36 mmHg.

**Figure 3:**
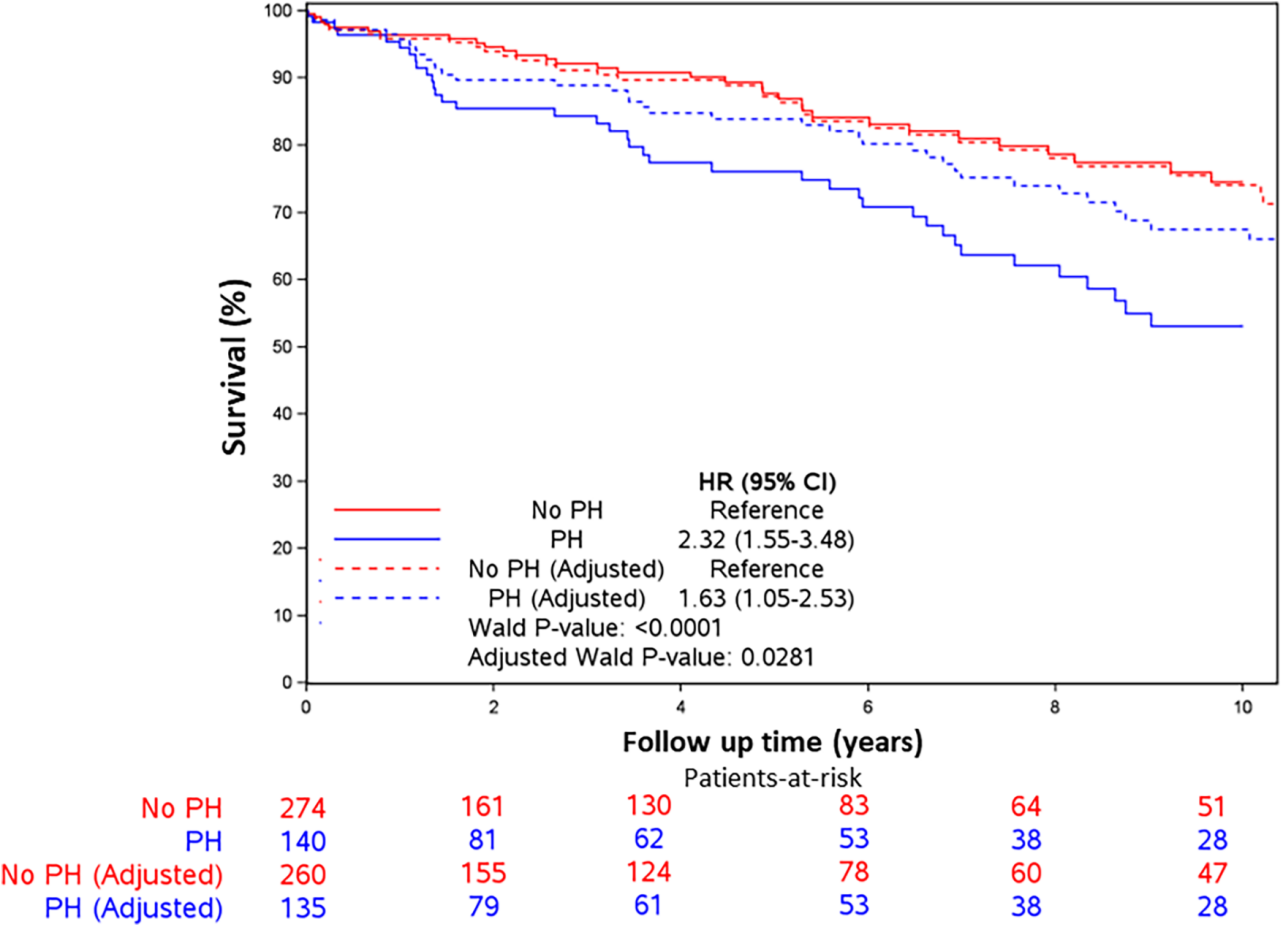
Kaplan–Meier survival curves. Kaplan–Meier survival curves for all-cause mortality by PH (PASP > 36 vs. <36 mmHg) in unadjusted and adjusted models. Patients with PASP > 36 mmHg had higher all-cause mortality in unadjusted models and after adjusting for age, sex, comorbidity index, and left ventricular filling pressures. Hazard ratios were 2.32 (95% CI 1.55, 3.48, *p* < 0.001) and 1.63 (95% CI 1.05, 2.53, *p* = 0.028), respectively.

Mortality was assessed over a median of 4.4 years (IQR: 0.1–10.7). The spline curves analysis showed that the risk of death started to rise continuously after the PASP reached a level between 35 and 36 mmHg for the whole cohort (Figure 2), and in males and females separately (Supplementary Figures S1A and B). Therefore, PASP of 36 mmHg was used as a cutoff, and PH (PASP > 36 mmHg) was associated with higher all-cause mortality in the univariate and multivariable analysis [HR for PH 1.63, 95% CI (1.05–2.53), *p* = 0.028] (Table 3 and Figure 3). Other independent factors associated with mortality were female sex [HR 1.88, 95% CI (1.20, 2.96), *p* = 0.006] and Charlson comorbidity index [HR 1.10, 95% CI (1.03, 1.18), *p* = 0.005] (Table 3). The correlation between PH and higher mortality was observed in both males and females (Supplementary Figures S2A, B). However, after adjusting for other factors, this correlation became non-significant (*p* = 0.08) for females.

**Table 3:** Multivariable analysis of factors associated with mortality.

Multivariable cox regression (*N* missing = 19)		
Variable	Hazard ratio (95% CI)	*p*-value
Pulmonary hypertension	1.63 (1.05, 2.53)	0.028
Age	1.01 (0.998, 1.03)	0.083
Female sex	1.88 (1.20, 2.96)	0.006
Charlson comorbidity index	1.10 (1.03, 1.18)	0.005
Medial E/e’ > 15	1.43 (0.88, 2.32)	0.148

E/e’, mitral early inflow diastolic/mitral annulus early tissue Doppler velocity.

PH, when present, was also found to be independently associated with symptoms (NYHA functional Class II–IV) [OR 2.28, 95% CI (1.40, 3.71), *p* < 0.001] in addition to the diagnosis of congestive heart failure [OR 5.01, 95% CI (2.30, 10.89), *p* < 0.001] (Table 4). PH was also independently associated with the presence of significant symptoms (NYHA Class III and IV) [OR 2.73 (1.60, 4.64), *p* < 0.001].

**Table 4:** Multivariable analysis of factors associated with symptoms (NYHA Class II–IV).

Variable	Multivariable logistic regression (*N* missing = 48)	
	Odds ratio (95% CI)	*p*-value
Pulmonary hypertension	2.28 (1.40, 3.71)	<0.001
Age	0.99 (0.98, 1.01)	0.394
Female sex	1.21 (0.77, 1.90)	0.406
Congestive heart failure	5.01 (2.30, 10.89)	<0.001
Charlson comorbidity index	0.99 (0.92, 1.06)	0.708

## Discussion

This study, which involved a large cohort of patients with ApHCM, has identified several significant findings: (1) PH as defined by a PASP value of > 36 mmHg on TTE was present in one-third of the patients, (2) female sex, atrial fibrillation, diagnosis of congestive heart failure, and elevated filling pressures were independently associated with PH in ApHCM patients, and (3) PH, when present, was associated with worse symptoms and higher all-cause mortality even after adjusting for known risk factors.

ApHCM is an understudied variant of HCM characterized by hypertrophy localized to the LV apex. It was previously considered “benign” due to the absence of obstruction, but studies have shown an increased risk of heart failure, arrhythmias, thromboembolic events, and death (4, 6, 7, 13). Although dynamic LV outflow tract obstruction is infrequent in ApHCM, elevated filling pressures due to diastolic dysfunction are frequently observed (7, 13–15) and serve as a mechanism for PH. In HCM, PH is thought to be post-capillary initially, and then over time, develop pre-capillary remodeling, leading to combined pre- and post-capillary PH (8, 9, 11). When present, PH is associated with worse outcomes in both patients with obstructive and non-obstructive HCM (8–11); however, there has been no specific research of the prevalence, risk associations, and implications of PH in ApHCM.

This study found a high prevalence of PH in up to one-third of patients with ApHCM. The results are similar to the reported prevalence rate of 38% in patients with other subtypes of HCM (predominantly septal, reverse-curve, neutral) using similar echocardiographic criteria (8). The factors associated with PH in ApHCM patients were found to be female sex, atrial fibrillation, diagnosis of congestive heart failure, and elevated filling pressures on echocardiogram, similar to what has been previously reported in non-selected HCM cohorts (8–10). The association between elevated filling pressures on echocardiogram (medial E/e') and a larger left atrial volume index, which reflects long-standing elevated left-sided filling pressures, suggests a group 2 mechanism of PH. Although a systematic approach to defining PH etiology was not performed in this retrospective assessment, a thorough chart review did not reveal alternative etiologies of PH. There are data showing worse outcomes in females with HCM (16, 17), and data herein clarify that this relationship persists in patients with ApHCM (7).

PH, when present, was independently associated with all-cause mortality, with other significant factors being female sex and Charlson comorbidity index. In the spline curve analysis, there was a continuous increase in the risk of mortality above a cutoff of PASP > 35–36 mmHg. This cutoff corresponds to a mean PA pressure of 25 mmHg, is recommended by the society guidelines for detecting PH with high sensitivity, and is previously shown to be associated with an increased risk of death in non-obstructive and obstructive HCMs without prior septal reduction treatment (8, 18, 19). PH has been shown to be associated with worse outcomes in left-sided heart disease including heart failure with a reduced and preserved ejection fraction and left-sided valve diseases (20). There are a few studies including one large study with over 1,500 patients showing worse outcomes associated with PH in patients with HCM (8–11), and to our knowledge, the current study is the first to systematically evaluate the prognostic significance of PH in patients with an apical subtype of HCM.

The novel myosin inhibitor, Mavacamten, was recently evaluated in a phase 2 trial (MAVERICK-HCM), which included a small group of patients with non-obstructive HCM (21). The study found that the treatment was well tolerated by the majority of patients and was associated with a reduction in cardiac biomarkers, indicating a decrease in wall stress. Mavacamten is currently under further investigation to assess improvements in clinical parameters (22). In addition, there is a need to explore its effects in patients with ApHCM.

## Limitations

Our study has several limitations—retrospective study design, single-center experience, and unavailability of TR jet to estimate PASP in approximately 25% of the patients from the original cohort which may change the true prevalence of PH in this population. The retrospective design limits the assessment of predictors, and only risk associations can be determined. The interobserver agreement of echocardiographic measurements is unavailable, but it is unlikely to affect the study results. Hemodynamic right heart catheterization, labs (autoimmune antibodies, NT-pro brain natriuretic peptide), pulmonary function tests, and ventilation perfusion scan were unavailable in most patients, which limits the exact characterization of the type (pre-capillary, post-capillary, or combined pre- and post-capillary) and etiology of PH (13). However, a systematic chart review of this large cohort did not reveal an obvious alternate etiology of PH in these patients. Some comorbidities, such as coronary artery disease and lung disease, could also potentially be associated with PH. However, the proportion of patients with these comorbidities was small, and their association with PH was not significant in the univariate analyses. Genetic testing for sarcomere mutations was not routinely available due to the retrospective nature of the study; therefore, genetic associations could not be tested.

## Conclusions

The present study shows a high prevalence rate of PH of 34% as determined by echocardiography in patients with ApHCM, with significantly higher prevalence in females vs. males (44% vs. 26%). The factors associated with PH were found to be female sex, atrial fibrillation, diagnosis of congestive heart failure, and elevated filling pressures on echocardiogram. PH when present was associated with both worse symptoms and higher all-cause mortality. There is a need for larger prospective studies to further evaluate the sex differences in PH, as well as the role of PH in categorizing risk and deciding the most suitable timing for intervention in these patients.

## Data Availability

The datasets presented in this article are not readily available until they receive approval from the institution and the IRB. Requests to access the datasets should be directed to the corresponding author.

## Supplementary material

Supplementary Figure 1 Spline curves for PASP cutoff for males and females. Spline curves demonstrate mortality risk across the range of measured PASP in **A**. Males **B**. Females. The risk increased for PASP > 35–36 mmHg in both males and females.

Supplementary Figure 2 Kaplan–Meier survival curves for males and females. Kaplan–Meier survival curves for all-cause mortality by PH (PASP > 36 vs. <36 mmHg) in unadjusted and adjusted models. Patients with PASP > 36 mmHg had higher all-cause mortality in unadjusted models and after adjusting for age, comorbidity index, and left ventricular filling pressures. Unadjusted and adjusted hazard ratios were 2.26 (95% CI 1.18,4.31, *p* = 0.013) and 2.03 (95% CI 1.03, 4.01, *p* = 0.040), respectively, for males and 1.91 (95% CI 1.10–3.32, *p* = 0.021) and 1.71 (95% CI 0.94, 3.11), *p* = 0.078), respectively, for females.
