# Supplementary material for: Prevalence, sex differences, and implications of pulmonary hypertension in patients with apical hypertrophic cardiomyopathy
Source: Front Cardiovasc Med. 2024 Jan 11;10:1288747. doi: 10.3389/fcvm.2023.1288747 (PMC10808763; doi:10.3389/fcvm.2023.1288747)
Supplement: Supplementary file 1 [file Table1.docx]

Supplemental Table 1. Baseline characteristics in males vs females

| **Variables** | **Total (N=414)** | **Males (N=233)** | **Females (N=181)** | **P-value** |
| --- | --- | --- | --- | --- |
| **Pulmonary hypertension** | 140 (33.8%) | 61 (26.2%) | 79 (43.6%) | <0.001 |
| **Age** | 59.4 (16.63) | 56.5 (16.53) | 63.3 (16.00) | <0.001 |
| **Race**, n (%) |  |  |  | 0.240 |
| White | 341 (82.4%) | 192 (82.4%) | 149 (82.3%) |  |
| Black | 20 (4.8%) | 9 (3.9%) | 11 (6.1%) |  |
| Asian | 17 (4.1%) | 11 (4.7%) | 6 (3.3%) |  |
| Choose Not to Disclose | 4 (1.0%) | 3 (1.3%) | 6 (3.3%) |  |
| Other | 9 (2.2%) | 4 (1.7%) | 0 (0.0%) |  |
| Unknown | 23 (5.6%) | 14 (6.0%) | 9 (5.0%) |  |
| **Body mass index** | 28.3 (5.20) | 28.8 (4.39) | 27.6 (6.02) | 0.019 |
| **Heart rate** (n=408) | 63.9 (11.97) | 61.8 (11.39) | 66.6 (12.20) | <0.001 |
| **Systolic blood pressure** (n=410) | 124.2 (19.41) | 124.0 (19.64) | 124.2 (19.41) | 0.857 |
| **Atrial fibrillation**, n (%) (n=396) | 58 (14.6%) | 25 (9.5%) | 33 (25.0%) | <0.001 |
| **Prior Myocardial infarction**, n (%) (n=395) | 21 (5.3%) | 11 (5.0%) | 10 (5.8%) | 0.717 |
| **Congestive heart failure**, n (%) (n=395) | 57 (14.4%) | 26 (11.7%) | 31 (17.9%) | 0.082 |
| **Ventricular tachycardia**, n (%) (n=395) | 34 (8.6%) | 24 (10.8%) | 10 (5.8%) | 0.077 |
| **Ventricular fibrillation**, n (%) (n=395) | 7 (1.8%) | 6 (2.7%) | 1 (0.6%) | 0.112 |
| **Cardiac Arrest**, n (%) (n=395) | 3 (0.8%) | 2 (0.9%) | 1 (0.6%) | 0.714 |
| **Premature ventricular contraction**, n (%) (n=395) | 29 (7.3%) | 20 (9.0%) | 9 (5.2%) | 0.150 |
| **Transient ischemic attack**, n (%) (n=395) | 22 (5.6%) | 11 (5.0%) | 11 (6.4%) | 0.546 |
| **Ischemic stroke**, n (%) (n=395) | 18 (4.6%) | 14 (6.3%) | 4 (2.3%) | 0.059 |
| **Hypertension**, n (%) (n=395) | 184 (46.6%) | 100 (45.0%) | 84 (48.6%) | 0.488 |
| **Diabetes**, n (%) (n=395) | 41 (10.4%) | 18 (8.1%) | 23 (13.3%) | 0.094 |
| **Chronic kidney disease**, n (%) (n=395) | 10 (2.5%) | 6 (2.7%) | 4 (2.3%) | 0.806 |
| **Lung disease**, n (%) (n=395) | 50 (12.7%) | 24 (10.8%) | 26 (15.0%) | 0.211 |
| **Coronary artery disease**, n (%) (n=395) | 39 (9.9%) | 20 (9.0%) | 19 (11.0%) | 0.514 |
| **Charlson Index, median (Q1, Q3)** (n=395) | 2 (0, 6) | 1 (0, 7) | 2 (1, 5) | 0.547 |
| **LV ejection fraction** | 66.8 (6.40) | 66.8 (5.80) | 66.8 (7.10) | 0.971 |
| **Diastolic dysfunction grade**, n (%) (n=126) |  |  |  | 0.119 |
| Normal | 14 (11.1%) | 9 (15.0%) | 5 (7.6%) |  |
| 1 (normal filling pressures) | 21 (16.7%) | 10 (16.7%) | 11 (16.7%) |  |
| 1a (mildly elevated filling pressures) | 13 (10.3%) | 5 (8.3%) | 8 (12.1%) |  |
| 2 (mild-moderated elevated filling pressures) | 32 (25.4%) | 17 (28.3%) | 15 (22.7%) |  |
| 3+ (severely elevated filling pressures) | 7 (5.6%) | 0 (0.0%) | 7 (10.6%) |  |
| Indeterminate | 39 (31.0%) | 19 (31.7%) | 20 (30.3%) |  |
| **Medial e’** (n=366) | 0.06 (0.07) | 0.07 (0.10) | 0.05 (0.03) | <0.001 |
| **Medial E/e’, median (Q1, Q3)** | 12 (10, 16) | 12 (10, 15) | 13 (10, 17) | 0.006 |
| **Medial E/e’>15**, n (%) | 108 (26.1%) | 50 (21.5%) | 58 (32.0%) | 0.015 |
| **E/A, median (Q1, Q3)** (n=340) | 1.3 (0.9, 1.8) | 1.4 (1.0, 1.8) | 1.0 (0.8, 1.6) | <0.001 |
| **RV S’** (n=200) | 0.12 (0.03) | 0.13 (0.03) | 0.12 (0.03) | 0.083 |
| **TAPSE** (n=59) | 20.0 (5.38) | 20.6 (4.49) | 19.3 (6.35) | 0.359 |
| **PASP** | 35.1 (10.56) | 33.09 (9.10) | 37.60 (11.74) | <0.001 |
| **PASP**, n (%) |  |  |  | <0.001 |
| <=36 | 274 (66.2%) | 172 (73.8%) | 102 (56.4%) |  |
| 37-60 | 130 (31.4%) | 58 (24.9%) | 72 (39.8%) |  |
| >60 | 10 (2.4%) | 3 (1.3%) | 7 (3.9%) |  |
| **>Mildly enlarged RV size**, n (%) (n=242) | 14 (5.8%) | 9 (6.9%) | 5 (4.5%) | 0.432 |
| **≥Mildly reduced RV function**, n (%) (n=73) | 16 (21.9%) | 10 (24.4%) | 6 (18.8%) | 0.363 |
| **Right atrial pressure, median (Q1, Q3)** (n=407) | 5 (5, 5) | 5 (5, 5) | 5 (5, 10) | 0.016 |
| **LA volume index** (n=349) | 41.3 (16.21) | 41.2 (14.26) | 41.4 (18.51) | 0.928 |
| **Max. instantaneous intracavitary gradient** (n=102) | 27.3 (16.75) | 26.5 (16.39) | 28.6 (17.45) | 0.554 |
| **≥ moderate Mitral Regurgitation**, n (%) (n=405) | 13 (3.2%) | 4 (1.8%) | 9 (5.1%) | 0.062 |

Abbreviations: LV, left ventricle; e’, mitral annulus early tissue Doppler velocity; E/e’, mitral early inflow diastolic/mitral annulus early tissue Doppler velocity; E/A, mitral inflow early/late diastolic velocity; s’, systolic tissue Doppler velocity; TAPSE, Tricuspid annular plane systolic excursion; PASP, pulmonary artery systolic pressure; RV, right ventricle; LA, left atrium
