# Supplementary material for: Prevalence, sex differences, and implications of pulmonary hypertension in patients with apical hypertrophic cardiomyopathy
Source: Front Cardiovasc Med. 2024 Jan 11;10:1288747. doi: 10.3389/fcvm.2023.1288747 (PMC10808763; doi:10.3389/fcvm.2023.1288747)
Supplement: Supplementary file 2 [file Table2.docx]

**Supplemental Table 2: Multivariable analysis of factors associated with PH (Model 2)**

*per unit change

|  | **Multivariable Logistic Regression (n missing=101)** | |
| --- | --- | --- |
| **Variable** | **Odds Ratio (95% CI)** | **p-value** |
| **Age** | 1.02 (1.00, 1.04) | 0.059 |
| **Female sex** | 3.32 (1.82, 6.06) | <0.001 |
| **≥ Moderate mitral regurgitation** | 3.30 (0.55, 19.90) | 0.194 |
| **Left atrial volume index*** | 1.08 (1.06, 1.11) | <0.001 |
| **Atrial fibrillation** | 0.54 (0.22, 1.31) | 0.171 |
| **Congestive heart failure** | 2.39 (1.04, 5.49) | 0.039 |
| **Charlson comorbidity index** | 1.07 (0.97, 1.18) | 0.194 |
