# Supplementary figures and images for: Prevalence, sex differences, and implications of pulmonary hypertension in patients with apical hypertrophic cardiomyopathy
Source: Front Cardiovasc Med. 2024 Jan 11;10:1288747. doi: 10.3389/fcvm.2023.1288747 (PMC10808763; doi:10.3389/fcvm.2023.1288747)

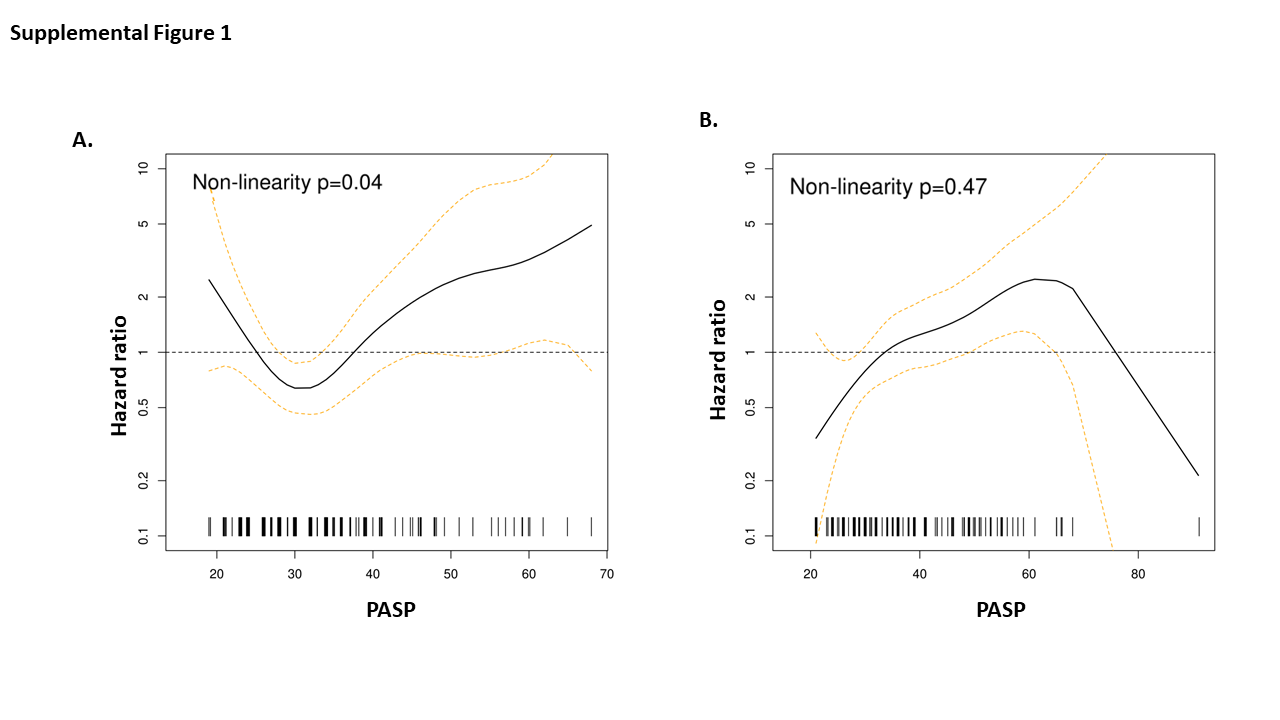

Supplement: Supplementary Figure 1 — Spline curves for PASP cutoff for males and females. Spline curves demonstrate mortality risk across the range of measured PASP in A. Males B. Females. The risk increased for PASP > 35–36 mmHg in both males and females. [file Image1.tif]

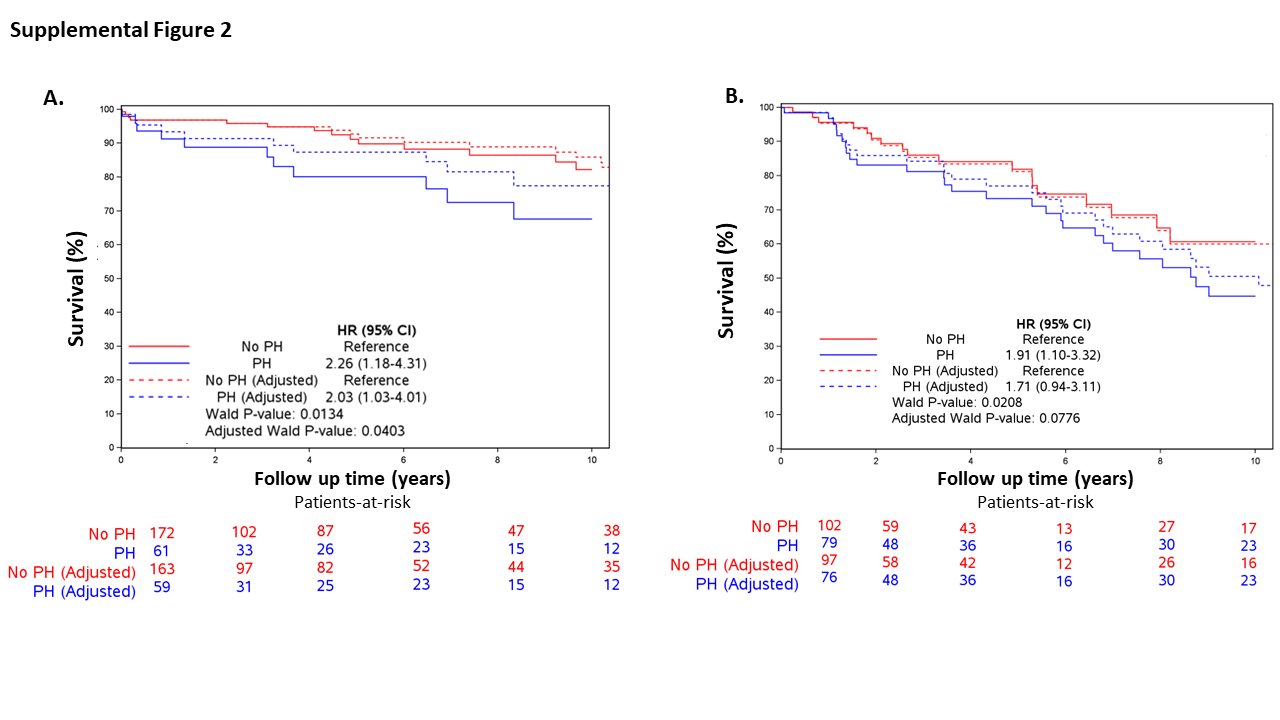

Supplement: Supplementary Figure 2 — Kaplan–Meier survival curves for males and females. Kaplan–Meier survival curves for all-cause mortality by PH (PASP > 36 vs. <36 mmHg) in unadjusted and adjusted models. Patients with PASP > 36 mmHg had higher all-cause mortality in unadjusted models and after adjusting for age, comorbidity index, and left ventricular filling pressures. Unadjusted and adjusted hazard ratios were 2.26 (95% CI 1.18,4.31, p = 0.013) and 2.03 (95% CI 1.03, 4.01, p = 0.040), respectively, for males and 1.91 (95% CI 1.10–3.32, p = 0.021) and 1.71 (95% CI 0.94, 3.11), p = 0.078), respectively, for females. [file Image2.tif]
